# A comparison of operative outcomes between standard and robotic laparoscopic surgery for endometrial cancer: A systematic review and meta‐analysis

**DOI:** 10.1002/rcs.1851

**Published:** 2017-08-01

**Authors:** Thomas Ind, Alex Laios, Matthew Hacking, Marielle Nobbenhuis

**Affiliations:** ^1^ Department of Gynaecological Oncology Royal Marsden Hospital London UK; ^2^ St George's University of London London UK

## Abstract

**Background:**

Evidence has been systematically assessed comparing robotic with standard laparoscopy for treatment of endometrial cancer.

**Methods:**

A search of Medline, Embase and Cochrane databases was performed until 30th October 2016.

**Results:**

Thirty‐six papers including 33 retrospective studies, two matched case–control studies and one randomized controlled study were used in a meta‐analysis. Information from a further seven registry/database studies were assessed descriptively. There were no differences in the duration of surgery but days stay in hospital were shorter in the robotic arm (0.46 days, 95%CI 0.26 to 0.66). A robotic approach had less blood loss (57.74 mL, 95%CI 38.29 to 77.20), less conversions to laparotomy (RR = 0.41, 95%CI 0.29 to 0.59), and less overall complications (RR = 0.82, 95%CI 0.72 to 0.93). A robotic approach had higher costs ($1746.20, 95%CI $63.37 to $3429.03).

**Conclusion:**

A robotic approach has favourable clinical outcomes but is more expensive.

## 1 INTRODUCTION

Evidence from randomised controlled trials support the use of laparoscopic techniques over open surgery for endometrial cancer.^1^ Standard laparoscopy for endometrial cancer is often possible but can be difficult to perform due to co‐morbidities such as obesity that can be associated with uterine malignancy.^2^ It has been proposed that robotic surgery is easier to learn than standard laparoscopy,^3^ and a number of studies have demonstrated improved ergonomics and outcomes *in vitro.* 3, 4 Furthermore, it has been suggested that the *in vitro* benefits for robotics might be paralleled by improved clinical outcomes for endometrial cancer patients. To date, a number of studies have demonstrated a higher proportion of women having a laparoscopic approach instead of open surgery when a robot is available.^5^, ^6^ Furthermore, they have suggested that this would improve the overall rate of conversion to laparotomy, operative complications and costs.^5^, ^6^ The aim of this study is to systematically assess comparative cohort studies from single institutions that compare standard laparoscopy with robot assisted laparoscopy for the treatment of endometrial cancer.

## 2 METHOD

A systematic search of Medline, Embase and the Cochrane database was performed for the period 1st January 1991 until 30th October 2016. No start date was used for the search. The search criteria included a search of titles, abstracts, and Medical Subject Headings for the words (‘uterine’ or ‘uterus’ or ‘endometrial’ or ‘endometrium’) and (‘carcinoma’ or ‘cancer’ or ‘neoplasia’ or ‘neoplasm’) and (‘robot’ or ‘robotic’ or ‘DaVinci’). Studies that compared a standard laparoscopic approach to endometrial cancer with a robotic approach within a discrete cohort were included. Papers were eliminated from the analysis if there was no such comparison or if it was not possible to extract data for endometrial cancer patients from other diagnoses. If two papers were published from the same institution, only the most recent manuscript was used to avoid duplication. The exception was when different outcomes were reported in separate papers. It was not possible to include papers that looked at outcomes from large registries as many patients from the other studies would have been included in national and regional databases resulting in duplication. However, registry papers were retrieved from the search and assessed descriptively in the discussion of this paper.

Data were taken from the text and tables of the published papers. The presentation of data depended on that reported in individual papers. For example, if a study reported both the pelvic and para‐aortic lymph node yields, it was only possible to include this data in total lymph node counts if that data was reported. A similar situation was applied to the reporting of operative complications. To avoid a complication being counted twice and potentially prejudicing one arm, a conversion to a laparotomy in it's own right was not reported in the complication fields but treated separately. The same applied to blood transfusions. Where possible, complications were reported as ‘total’ but divided into ‘major’ and ‘minor’ in nature if reported as well as ‘intra‐operative’ and ‘post‐operative’ if separated in a paper's text. If the Clavien‐Dindo classification was used in a paper, post‐operative complications classed as III or above were defined as ‘major’. Additional information clarifying data was sought from three authors and in one case this was provided.^7^

Costs and charges were presented in United States Dollars. If this was reported in another currency then this was converted to Dollars using the exchange rate published for the middle year of the recruitment period from the Bank of England website (www.bankofengland.co.uk). The data were recorded using Review Manager.^8^ Dichotomous data were presented as Risk Ratios using the Mantel–Haenszel method with random effects.^9^ Continuous data were presented as means with standard deviations and analysed using the Inverse Variance method using random effects.^10^ When continuous data were presented as medians with ranges, the data were converted for inclusion into the meta‐analysis using the method described by Hozo *et al..* 11 When only interquartile ranges were reported, the data could not be included into the meta‐analysis.

## 3 RESULTS

**Figure 1:**
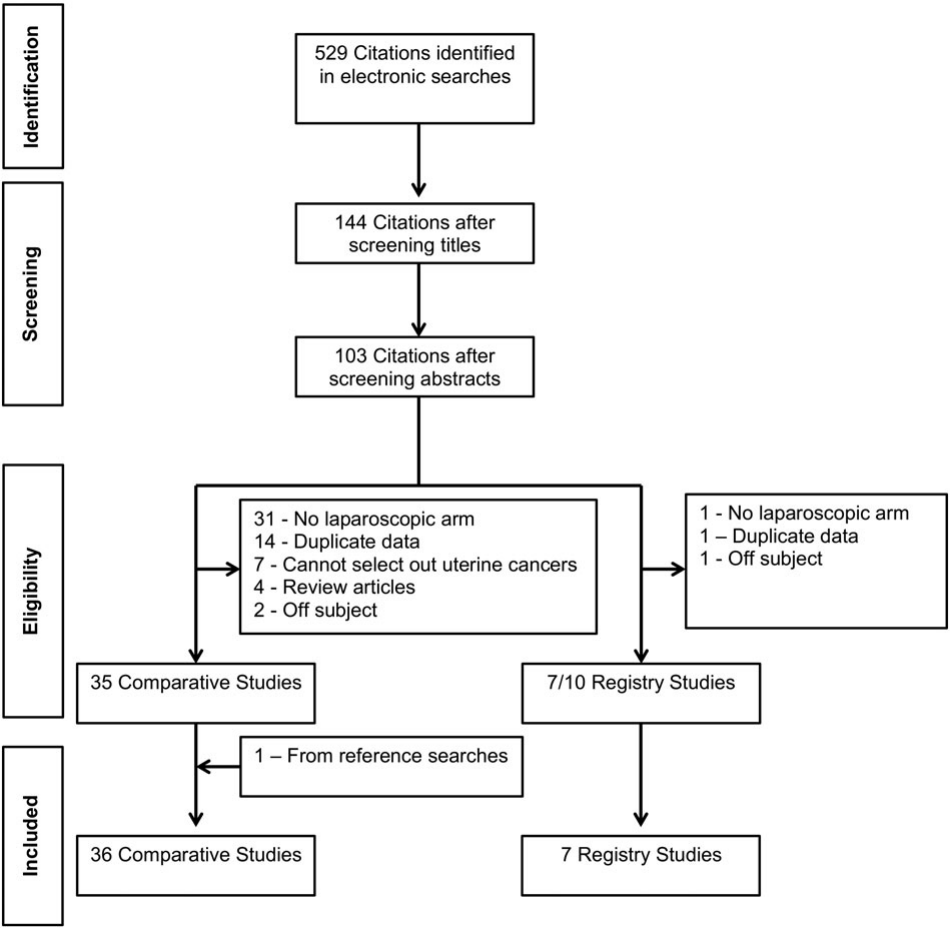
PRISMA flow chart for trial identification and selection

A flowchart of how papers were selected is given in Figure 1. This revealed 35 papers that were included in the study.^5^, ^12^, ^13^, ^14^, ^15^, ^16^, ^17^, ^18^, ^19^, ^20^, ^21^, ^22^, ^23^, ^24^, ^25^, ^26^, ^27^, ^28^, ^29^, ^30^, ^31^, ^32^, ^33^, ^34^, ^35^, ^36^, ^37^, ^38^, ^39^, ^40^, ^41^, ^42^, ^43^, ^44^ A further hand‐search of review article references included one additional paper.^45^ Therefore, a total of 36 papers were included in the analysis and these involved 8075 patients (3830 robotic and 4245 laparoscopic). A list of papers included in the meta‐analysis and the outcomes included are detailed in Table 1. This included 35 retrospective cohort studies of which two contained matched case‐controls.^19^, ^31^ In addition, there was one randomised controlled study^32^ (Table 1). Furthermore, seven papers reporting data from registries were carefully read and used for comparative discussion in the relevant section of this paper.^46^, ^47^, ^48^, ^49^, ^50^, ^51^

**Table 1:** Studies selected for inclusion into the meta‐analyses

First author	Year	Design	Countries	Period of recruitment	N‐rob	N‐lap	Outcomes included in meta‐analysis
Bell	2008	RCC	USA	May 2000 to Jun 2009	40	30	OT, Los, RNA, TLN, BL, BT, ac, AMC, C, Tc
Boggess	2008	RCC	USA	Jun 2005 to Dec 2007 – Rob Apr 2000 to Sep 2004 ‐ lap	103	81	OT, Los, BL, BT, TLN, PLN, PALN, cl, ac, AIC, mic, APC
Hoekstra	2009	RCC	USA	Jul 2007 to Jul 2008	24	7	BT, cl, Ra, ac, AMC, AIC
Seamon	2009	RCC	USA	Jan 2006 to Apr 2008	105	76	OT, ort, Orit, Los, BT, PLN, PALN, BL, cl, ac
Holtz	2010	RCC	USA	Jul 2007 to Jul 2008	13	20	OT, LOS, PLN, PALN, BL, DHb, CL, AC, AMC, AIC, MIC, APC, MPC, TC
Jung	2010	RCC	Korea	May 2006 to Jan 2009	28	25	OT, Los, PLN, PALN, BT, cl, ac, AMC, AIC, mic, APC, MPC
Lim	2011	mRCC	USA	Mar 2008 to Jul 2010	122	122	OT, Los, TLN, PLN, PALN, BL, BT, cl, Ra, ac, AMC, AIC, mic
Magrina	2011	RCC	USA	Mar 2004 to Dec 2007 ‐ rob; Nov 1999 to Aug 2006 ‐ lap	37	67	OT, LOS, PLN, PALN, BL, BT, CL, RA, AC, AIC, APC, rec
Martino	2011	RCC	USA	Sep 2005 to Jun 2010	101	114	PPS
Shah	2011	RCC	USA	Jan 2009 to Dec 2009	43	118	OT, Los, BL, cl, ac, AIC, mic, APC
Coronado	2012	RCC	Spain	2003 to Jun 2011	71	84	OT, LOS, PLN, BL, BT, DHb, CL, AC, AIC, MIC, APC, TC
Escobar	2012	mRCC	USA	Apr 2009 to Sep 2010	30	60	OT, Los, PLN, PALN, BL, BT, cl, ac, AMC, AIC, mic
Estape	2012	RCC	USA	2002 to 2009; robot from 2006	102	104	OT, Los, TLN, BL, BT, cl, RI, Ra, ac, AMC, AIC, mic
Fagotti	2012	RCC	Italy	Feb 2009 to Jun 2011	75	75	OT, Los, TLN, BL, cl, ac, AMC, AIC, APC, MPC
Fleming	2012	RCC	USA	Jun 2008 to Sep 2010	23	43	OT, Los, ort, PLN, PALN, BL, cl, ac, AMC, AIC, mic, MPC, PPS, INU, PNU
Leitao MM Jr	2012	RCC	USA	May 2007 to Dec 2010	347	302	OT, ort, Los, BT, TLN, PLN, PALN, BL, cl, ac, AMC, APC
Nevadunsky	2012	RCC	USA	Aug 2006 to Jan 2009	102	115	OT, Los, BL, BT, cl, ac, APC
Venkat	2012	RCC	USA	2008–2010	27	27	OT, ort, Los, TLN, BL
Cardenas‐Goicoechea	2013	RCC	USA	Dec 2007 to Apr 2010 – Rob Jan 2003 to Dec 2007 ‐ lap	187	245	OT, Los, TLN, PLN, PALN, BL, BT, cl, RI, Ra, ac, AIC, mic, APC
Desille‐Gbaguidi	2013	RCC	France	2008 to Dec 2011	20	15	OT, Los, TLN, BL, Ra, Tc
Leitao MM Jr	2013	RCC	USA	May 2007 to Jun 2010	239	236	PPS, D1PS
Turunen	2013	RCC	Finland	May 2009 to Feb 2013	67	150	OT, PLN, BL, cl
Leitao MM Jr	2014	RCC	USA	Jan 2009 to Dec 2010	262	132	TC
Mendivil	2014	RCC	USA	Sep 2008 to Dec 2011	13	16	OT, Los, TLN, BL, BT, cl, Ra, ac, AIC, mic, APC
Pakish	2014	RCC	USA & Brazil	Jan 2007 to Nov 2012	52	142	OT, PLN, PALN, BL, BT, cl, Ra, AIC, mic
Seror	2014	RCC	France	Jan 2002 to Dec 2011. (robotics started in 2008)	40	106	BT, cl, ac, AMC, AIC, mic, APC, MPC
Chiou	2015	RCC	Taiwan	2011 to 2013 ‐ rob; 2005–2013 ‐ lap	86	150	OT, Los, DFD, TLN, PLN, BL, ac, AMC, PPS, D1PS
Corrado	2015	RCC	Italy	Jan 2001 to Dec 2013	72	277	OT, LOS, PLN, BL, BT, CL, RI, AC, AMC, AIC, MIC, APC, MPC, rec
Frey	2015	RCC	UA	May 2006 to Oct 2010	77	45	OT, Los, TLN, PLN, PALN, BL, cl
Ind	2015	RCC	UK	Jan 2010 to Dec 2013; (robot from 2012)	24	77	OT, LOS, BL, BT, DHb, CL, AL, AC, AMC, AIC, MIC, APC, MPC, TC
Manchana	2015	RCC	Thailand	Jan 2011 ro Dec 20014	28	47	BT, cl, AIC, mic, APC
Turner	2015	RCC	USA	Jan 2008 to may 2012	122	213	Ort, BL, cl, INU, PNU
Barrie	2016	RCC	USA	Jan 2009 to Jan 2014	745	688	Ac, AMC, AIC, mic, APC, MPC, cl, BT
Johnson	2016	RCC	USA	Oct 2008 to Sep 2012	353	187	OT, ort, Los, PLN, PALN, BL, cl, Ra, ac, AIC, APC
Maenpaa	2016	RCT	Finland	Dec 2010 to Oct 2013	50	49	OT, ORT, LOS, TLN, PLN, BL, BT, PHb, DHb, CL, AC, AMC, AIC, MIC, APC, MPC, D1PS, D2PS
Pilka	2016	RCC	Czech Republic	Oct 2012 to Jun 2015	64	13	TLN, BL, DHb, PPS

Abbreviations

RCC – Retrospective Cohort Comparison, mRCC – Matched Retrospective Cohort Comparison, RCT – Randomised Controlled Trial

OT – Operative Time; ORT ‐ Operating Room Time; LOS – Length Of Stay; ORIT – Operating Room to Incision Time; DFD – Days to Full Diet; RNA – Days Return to Normal Activity

TLN – Total Lymph Node count; PLN – Pelvic Lymph Node count; PALN – Para‐Aortic Lymph Node count

BL – Blood Loss; BT – Blood transfusion; PHb – Post‐operative Haemaglobin, DHb – Drop in Haemaglobin

CL – Conversion to Laparotomy; RI – Re‐Intervention; RA – Re‐Admisssion

AC – All Complications; AMC – All Major Complications; AIC – All Intra‐operative complication; MIC – Major Intra‐operative complications; APC – All Post‐operative complications; MPC – Major Post‐operative Complications

PPS – Post‐operative Pain Score; D1PS – Day 1 Pain Score; D2PS – Dat 2 Pain Score; INU – Intra‐operative Narcotic Usage; PNU; Post‐operative Narcotic Usage

C‐ Charges; TC – Total Costs

Rec ‐ Recurrences

**Figure 2:**
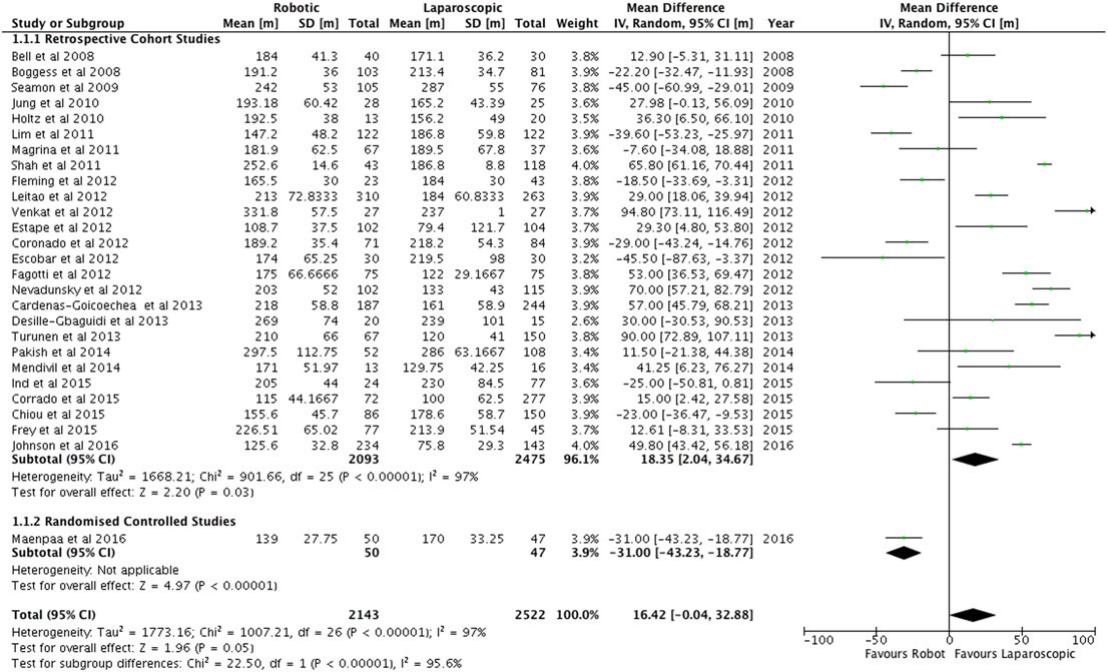
Duration of operations for endometrial cancer (mins)

**Figure 3:**
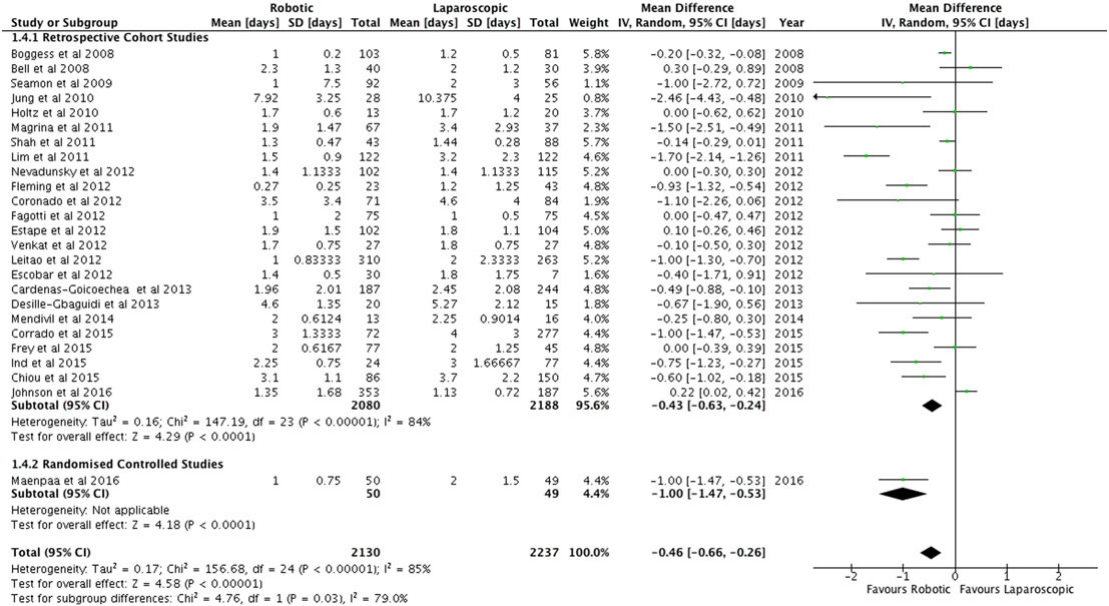
Days in hospital following surgery for endometrial cancer

A summary of the outcomes is shown in Table 2. Across all studies, there was no statistically significant difference in the duration of surgery or operating room times (Table 2). However, the one randomized controlled study reported shorter operating times (Figure 2) and total operating room times for robotic surgery.^32^ This contrasted with the retrospective cohort studies that reported a longer operating time of 18.4 minutes (95%CI = 2.0–34.7 min) for the robotic arm (Figure 2) but no difference in the total operating theatre time (Table 2). One study reported a longer time from arrival in theatre to the surgical incision for robotic surgery (Table 2).^39^ The number of days stay in hospital was shorter in the robotic arm compared with standard laparoscopy (Figure 3).

**Table 2:** Studies, participants and outcomes in a meta‐analysis comparing robotic to standard laparoscopy for endometrial cancer – Summary of 36 studies

Outcome or subgroup	Studies	Participants	Statistical method	Effect estimate
**Operation and hospital durations**				
Operation time (m)	27	4665	Mean difference (IV, random, 95% CI)	16.42 (−0.04, 32.88)
Operating room time (m)	7	1647	Mean difference (IV, random, 95% CI)	17.76 (−15.09, 50.61)
In OR to incision time (m)	1	181	Mean difference (IV, random, 95% CI)	6.00 (2.80, 9.20)
Hospital stay (days)	25	4367	Mean difference (IV, random, 95% CI)	−0.46 (−0.66, −0.26)*
Receiving full diet (days)	1	236	Mean difference (IV, random, 95% CI)	−0.20 (−0.35, −0.05)*
Days return to normal activity (days)	1	70	Mean difference (IV, random, 95% CI)	−7.50 (−12.04, −2.96)*
**Lymph nodes**				
Total lymph node count (n)	14	2086	Mean difference (IV, random, 95% CI)	−0.14 (−5.73, 5.46)
Pelvic lymph node count (n)	18	2852	Mean difference (IV, random, 95% CI)	1.24 (−0.75, 3.22)
Para‐aortic lymph node count (n)	13	1908	Mean difference (IV, random, 95% CI)	0.83 (−1.04, 2.71)
B**leeding**				
Blood loss (ml)	28	5115	Mean difference (IV, random, 95% CI)	−57.74 (−77.20, −38.27)*
Blood transfusions	21	4911	Risk ratio (M‐H, random, 95% CI)	0.77 (0.5, 1.07)
Postoperative Haemoglobin (g/L)	1	99	Mean difference (IV, random, 95% CI)	−5.00 (−10.77, 0.77)
Drop in Haemoglobin (g/L)	5	457	Mean difference (IV, random, 95% CI)	−3.93 (−8.72, 0.87)
**Adverse events**				
Conversion to laparotomy	28	6558	Risk ratio (M‐H, random, 95% CI)	0.41 (0.29, 0.59)*
Re‐operation/re‐intervention	3	594	Risk ratio (M‐H, random, 95% CI)	0.78 (0.02, 30.03)
Re‐admission	9	1823	Risk ratio (M‐H, random, 95% CI)	1.55 (0.82, 2.92)
All complications	25	5823	Risk ratio (M‐H, random, 95% CI)	0.82 (0.72, 0.93)*
All major complications	16	3787	Risk ratio (M‐H, random, 95% CI)	1.06 (0.61, 1.90)
Intra‐operative complications	22	4853	Risk ratio (M‐H, random, 95% CI)	0.81 (0.61, 1.06)
Major intra‐operative complication	18	3957	Risk ratio (M‐H, random, 95% CI)	0.85 (0.58, 1.23)
Post‐operative complications	18	4327	Risk ratio (M‐H, random, 95% CI)	0.85 (0.72, 1.02)
Major post‐operative complications	9	2430	Risk ratio (M‐H, random, 95% CI)	1.18 (0.79, 1.76)
**Pain and analgesia**				
Postoperative visual analogue pain score (0–10)	5	1070	Mean difference (IV, random, 95% CI)	−0.08 (−0.36, 0.20)
Day 1 visual analogue pain score (0–10)	3	788	Mean difference (IV, random, 95% CI)	−0.48 (−1.07, 0.10)
Day 2 visual analogue pain score (0–10)	1	27	Mean difference (IV, random, 95% CI)	0.00 (−1.31, 1.31)
Intra‐operative narcotic usage (mg m‐e)	2	179	Mean difference (IV, random, 95% CI)	−40.00 (−52.13, −27.87)
Post‐operative narcotic usage (mg m‐e)	2	180	Mean difference (IV, random, 95% CI)	−1.50 (−8.83, 5.82)
**Finances**				
Charges ($)	1	70	Mean difference (IV, random, 95% CI)	1746.20 (63.37, 3429.03)*
Total costs ($)	6	788	Mean difference (IV, random, 95% CI)	1869.42 (267.89, 3470.94)*
**Oncological Ourtomes**				
Recurrences	2	453	Risk ratio (M‐H, random, 95% CI)	0.66 (0.33, 1.34)

* = Statistically Significant

IV = Inverse Variance

M‐H = Mantel–Haenzel

There was no difference in the total number of lymph nodes removed in the two arms (Table 2). Furthermore, there were no differences between the pelvic and para‐aortic lymph node yields when analysed separately (Table 2).

**Figure 4:**
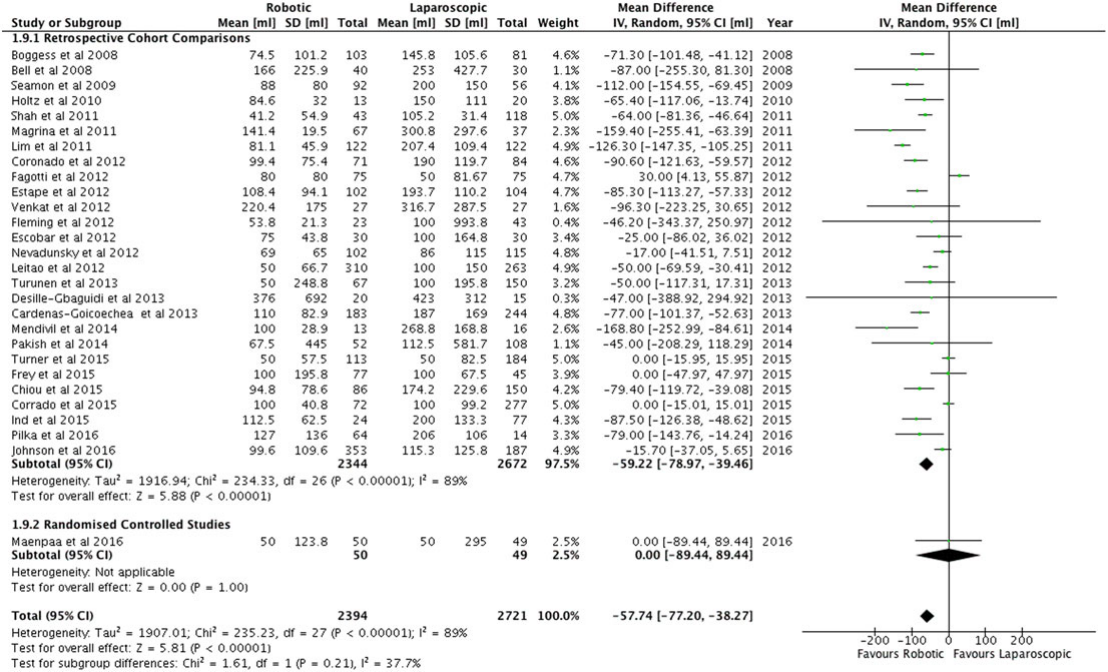
Mean estimated blood loss (mL) following surgery for endometrial cancer

**Figure 5:**
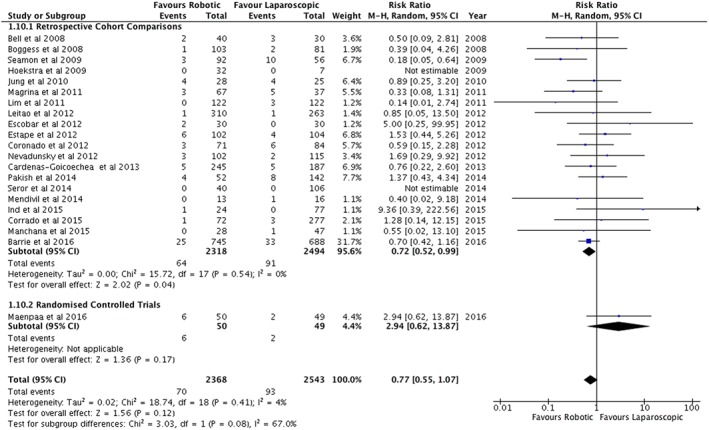
Blood transfusions following surgery for endometrial cancer

The estimated blood loss was on average 57.7 mL less during robotic surgery (95%CI 38.3 to 77.2) (Figure 4). This difference was not reflected in the use of blood transfusions, which was significantly less for robotic surgery in the retrospective studies but not in the randomized controlled study nor in a meta‐analysis of all the papers (Figure 5). No differences were found in the post‐operative haemoglobin nor in the post‐operative drop in haemoglobin concentration (Table 2).

**Figure 6:**
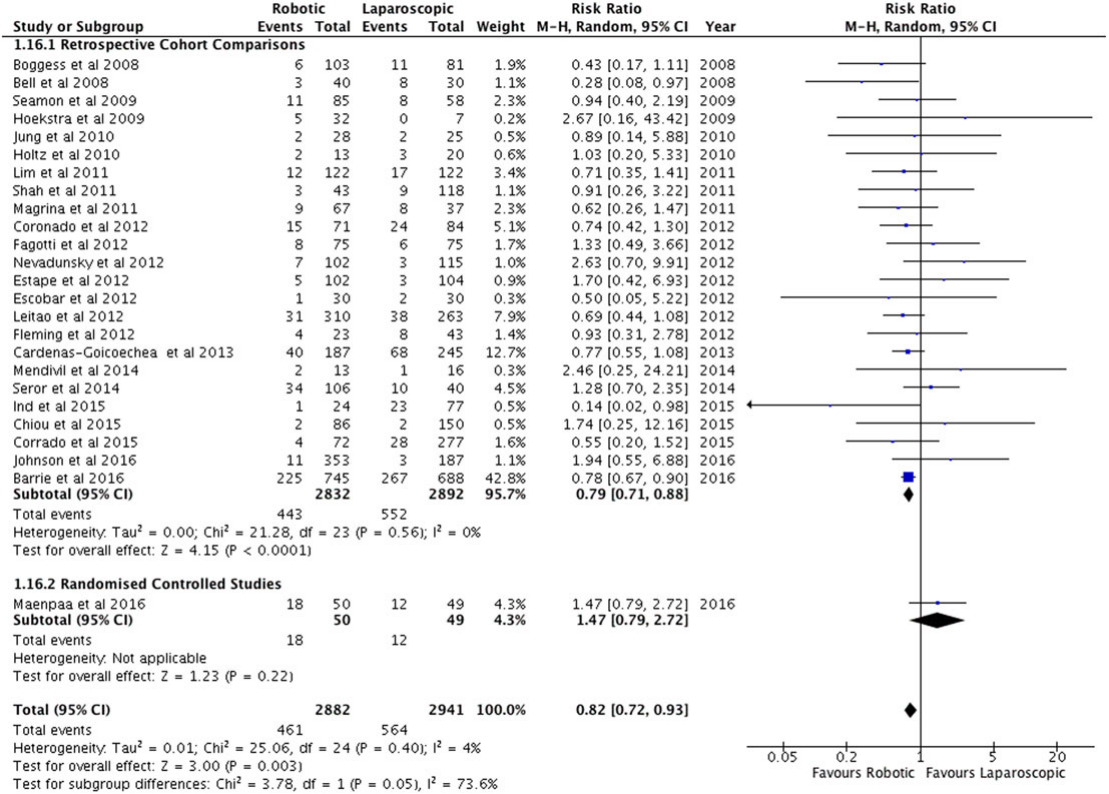
All complications related to surgery for endometrial cancer

**Figure 7:**
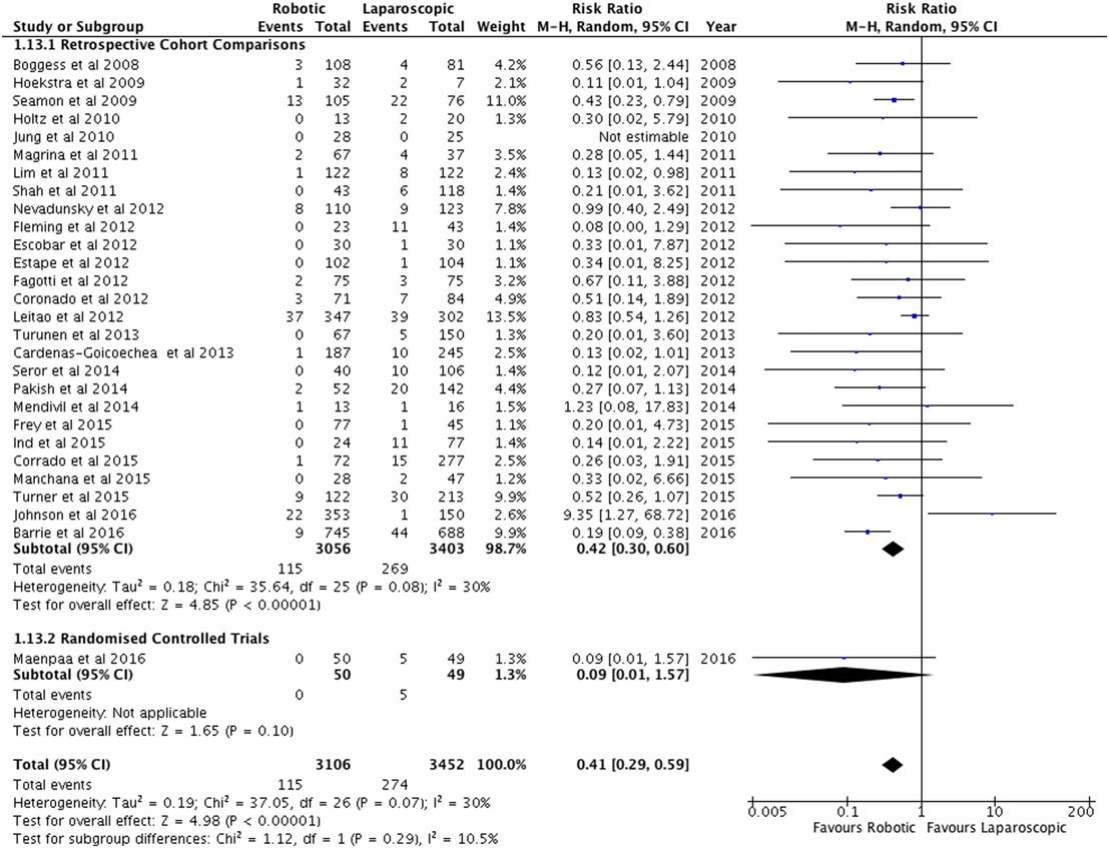
Conversions to laparotomy following surgery for endometrial cancer

For adverse outcomes, significant differences were not found for re‐interventions, re‐admissions, major complications, intra‐operative complications, major intra‐operative complications, post‐operative complications or major post‐operative complications (Table 2). However, there were less total complications in the robotic arm (RR = 0.82, 95%CI = 0.72 to 0.93) (Figure 6). Furthermore, there were significantly less conversions to laparotomy for robotic surgery compared with standard laparoscopy (RR = 0.41, 95%CI = 0.29 to 0.59) (Figure 7).

No differences could be demonstrated between the two groups for pain scores or post‐operative analgesia usage (Table 2). However, data from two studies showed significantly less intra‐operative narcotic analgesia usage in the robotic group (−40 mg morphine equivalents, 95%CI = −52.11 to −27.85 mg) although this was heavily weighted by one study.^22^ No differences were demonstrated in the risk of recurrence (Table 2).

Six studies reported the total costs of surgery and could be used in a meta‐analysis. All but one showed an increased cost with the robotic arm with a mean additional cost of $1869.42 (95%CI = $267.89 to $3470.94).

## 4 DISCUSSION

These data are favourable towards the robotic arm for hospital stay, return to normal activity, return to a normal diet, conversion to laparotomy, operative complications and blood loss. The total cost is in favour of standard laparoscopy. All but three studies assessed are retrospective cohort reviews, two are matched retrospective reviews and one a randomized controlled study (Table 2). Therefore, the quality of the evidence is low although it is bolstered by large numbers of papers and patients. One criticism is that in many of the papers, the robotic arm consists of an early series for the surgical teams. Outcomes with robotic surgery improve with numbers performed^29^, ^52^ so this would potentially be biasing the results in favour of the more established standard laparoscopy arm. Furthermore, some authors have acknowledged worse co‐morbidity in the robotic arms^5^, ^45^ of their studies with obesity in particular associated with worse outcomes.^53^ Therefore the data in favour of robotic laparoscopy is in spite of adverse confounders.

Other recent reviews and meta‐analyses of the subject exist.^54^, ^55^ They do not include all the citations that are in this study nor the randomized controlled study. Some of these meta‐analyses include registry studies even though some analyse the same databases and include patients reported in the institutional cohorts. However, the findings of less operative conversions, lower blood loss, and a shorter hospital stay are consistent findings within meta‐analyses but this study also demonstrates less overall complications in the robotic arm as well as higher costs.^54^, ^55^

This study found significantly longer operating times for robotic surgery in the retrospective cohort studies. However, the one randomized controlled study showed shorter operating times for robotic surgery.^32^ This may be due to the ‘early series’ effect described when a teams first few operations took longer than the later procedures in their series but in one study where the surgeon and team was already experienced in robotic surgery, longer operating times were still demonstrated.^5^ It is possible that this is a power effect and a larger study with even more numbers would have demonstrated a longer duration of surgery. From studies reporting outcomes from registries and databases, one study reported a non‐significant shorter operative time in the robotic arm and no studies report longer operative times.^46^ The mean difference of 18 minutes has to be put in perspective as most people accept the benefits of laparoscopic compared to open surgery for endometrial cancer.^1^, ^56^ A meta‐analysis has shown that a standard laparoscopic approach has an additional operative duration of 33 minutes over laparotomy.^56^

This study demonstrates a shorter hospital stay for robotic cases. This is supported by one registry study that showed a significantly lower proportion of women staying three nights or more in hospital.^51^ One other registry study reports a non‐significant shorter stay in the robotic group.^46^ Return to normal activity is shorter for robotics in the one study that reports this outcome in the meta‐analysis.^12^ One registry study reports on this.^46^ That study^46^ reports on a 6.7 days quicker return to normal activity for the robotic arm but reports this as being non‐significant. However, using the Inverse Variance method this would have 95% confidence intervals of 2.05 to 11.35 days shorter return to normal activity which supports the data we report. The reduction in conversion to laparotomies and less complications might explain these findings as one would expect a patient who had a laparotomy or one who suffered complications to spend longer in hospital and take longer to return to normal activity.

In this analysis we demonstrated less blood loss in the robotic arm. However, this could be perceived as a surrogate outcome as 50 mL less blood loss might not be reflected in a drop in haemoglobin concentration or the use of blood transfusions. Although blood transfusion usage was much lower in the robotic arm (RR = 0.76, 95%CI 0.57 to 1.01) this failed to reach statistical significance. Furthermore, no difference in the drop in haemoglobin could be demonstrated either. Blood loss was reported in one registry study and was not significantly different.^46^ Blood transfusion usage was not shown to be different in any of the registry studies but was lower in all four papers that reported this outcome.^46^, ^49^, ^50^, ^51^ Therefore, the importance or not in the finding of 50 mL less blood loss remains to be defined.

The finding of less conversions to laparotomy is an important one as the relative risk is 0.42 with tight confidence intervals (0.30 to 0.59). This is likely to be related to the increased ergonomics of robotic surgery over standard laparoscopy.^57^ However, the outcome is not supported in a registry study.^51^ Re‐operation and re‐admission rates are also reported in registry studies without any demonstrable significant difference.

The findings of less overall complications may also be related to ergonomic reasons although it will be interesting to see with time how further studies not influenced by the ‘early series’ effect will alter the analysis of intra‐operative, post‐operative, and major complications. The registry studies have conflicting results for this outcome. Total complication rates are very heterogeneous as they are dependent on the definition of a complication and the systematic way in which complications are collected. One registry study reported ‘similar morbidity’ yet the analysis in a table showed significantly less medical complications, significantly less bladder injuries, and significantly less re‐operations for robotic surgery compared with standard laparoscopy.^50^ Another study by the same group showed a 4% increase in all complications and medical complications in the robotic arm.^49^

The cost analysis is in favour of the standard laparoscopy arm of the study being $1869.42 less expensive. This is consistent with outcomes from a large registry study where standard laparoscopy was $1291.00 cheaper than a robotic approach to endometrial cancer.^50^ This figure reduces to $688.00 for individual surgeons who perform more than 50 cases a year^48^ and that caseload could be considered as an absolute minimum for endometrial cancer surgeons. Other studies that report on hospital charges rather than costs show greater differences.^51^, ^58^ However, some might argue that such an increased cost compares favourably compared with other interventions in the field of gynecological oncology such as some chemotherapy agents. What a straight comparison between robotic and standard approaches does not reveal is the additional cost from those patients who have open surgery in institutions not using robotics. To date, two studies have demonstrated greater utilisation of laparoscopic approaches with the use of the robot with less laparotomies, less complications and less overall costs when including the expense of open surgery into the cohorts.^5^, ^6^ One problem with analysing cost data in such a way is that different countries have variable healthcare reimbursement systems and wage costs. For example in some countries where there is social healthcare, surgeons are salaried by institutions and in other countries they charge separately. Therefore, a cost–benefit may exist in one healthcare system and not in another and it is difficult to interpret how this data would apply to a single institution although it is clearly of interest.

One matter to consider when assessing these outcomes is the innovation in new platforms over time. In early series, the Da‐Vinci Standard® system will have been used, whereas in latter series the fourth generation of platform (DaVinci Xi®) may have been available. To date there is no published data on the value of the updated systems on outcomes and it would be interesting to analyse this. Furthermore, different institutions have different protocols for para‐aortic and pelvic lymph node dissections resulting in a heterogeneity of operations performed across institutions. If a consensus ever occurs on the role of lymphadenectomy in endometrial cancer then it would be wise to assess separate subgroups but this is not possible currently.

In summary, this study demonstrates that the current evidence is in favour of robotic assisted laparoscopy for endometrial cancer over standard laparoscopy for clinic outcomes but costs are probably greater. To date there are only 99 patients recruited to randomized controlled trials^32^ and an increase in this number will undoubtedly provide stronger evidence.

## CONFLICT OF INTERESTS

Marielle Nobbenhuis and Thomas Ind have proctored for Intuitive Surgical.

## ETHICS

As this is a review no ethics was required.
